# *Leucothoe vaderotti*, a new Atlantic *Leucothoe* (Crustacea, Amphipoda) belonging to the “*spinicarpa*-clade” (Crustacea, Amphipoda)

**DOI:** 10.3897/zookeys.731.19813

**Published:** 2018-01-23

**Authors:** Traudl Krapp-Schickel

**Affiliations:** 1 Forschungsmuseum A. Koenig, Adenauerallee 160, D-53113 Bonn, Germany

**Keywords:** Amphipoda, Atlantic Ocean, IceAGE, Leucothoidae, new species, taxonomy

## Abstract

Within the international IceAGE project (Icelandic marine Animals: Genetics and Ecology) some leucothoid amphipods (Crustacea) were collected, among them a rather small new species, belonging to the “*Leucothoe spinicarpa*-clade.”

## Introduction

Within the IceAGE-collections (a follow-up of BIOICE, see for example Berge and Vader, 1997) some rather small specimens of the genus *Leucothoe* were collected, similar to the ones described in Krapp-Schickel and Vader (2012) from the Norwegian Sea, called “Leucothoe aff. spinicarpa”. This genus is extremely easy to recognize as such, but its many species are differentiated from each other only by subtle characters, which has traditionally led to a wholesale lumping of many species under just a few names, especially *Leucothoe spinicarpa* (Abildgaard, 1789). In the last years, it has become clear that the genus, with many of its species living commensally, is a very speciose one and there is no doubt a considerable number of as-yet undescribed species to be discovered (cf. Thomas and Klebba 2007).

## Material and methods

Samples were taken during IceAGE1 and IceAGE2 (with research vessels ‘Meteor’ and ‘Poseidon’; see Brix et al. 2014); they were fixed in cold 96% un-denaturated ethanol, sorted on ice, and stored at 0–4 °C after sorting. They were identified, some mounted on slides with Faure’s fluid, and drawn using a Leitz Laborlux microscope. “Inking” was done with a Wacom tablet, following Coleman, 2003.

Acronyms used in the morphological descriptions are as follows:

**A 1, 2** antenna 1, 2

**Md** mandible

**acc.** accessory

**Mx 1, 2** maxilla 1, 2

**ad.** adult

**Mxp** maxilliped

**art** article

**OP** outer plate

**Cx** coxal plate

**P 3–7** peraeopod 3–7

**Ep** epimeral plate

**Ped** peduncle

**flag** flagellum

**Pl** pleopod

**Gn 1, 2** gnathopod 1, 2

**T** telson

**Hd** head

**U 1–3** uropod 1–3

**IP** inner plate

**UL** upper lip

**LL** lower lip

## Results

### Leucothoidae Dana, 1852

#### Genus *Leucothoe* Leach, 1814

##### Leucothoe vaderotti sp. n.

Taxon classificationAnimaliaEricalesEricaceae

http://zoobank.org/BB292914-F4F1-451F-829A-4010FBCF5C31

Figs 1 , 2

Leucothoe aff. spinicarpa Krapp-Schickel & Vader, 2012, 386–388, fig. 3, 4

###### Material.

*Holotype* DZMB–HH 56285 (ZMH K–46787): 1 ad. 5.5 mm, 61°59.83'–61°59.26 N, 000°30.40'–000°32.32'E, Poseidon IceAGE 2, POS 456, 24.7.2013, Norwegian Channel, North Sea, St. 867, 302.5–290 m depth, EBS Supra, 300 μm. In alcohol.

**Figure 1.**
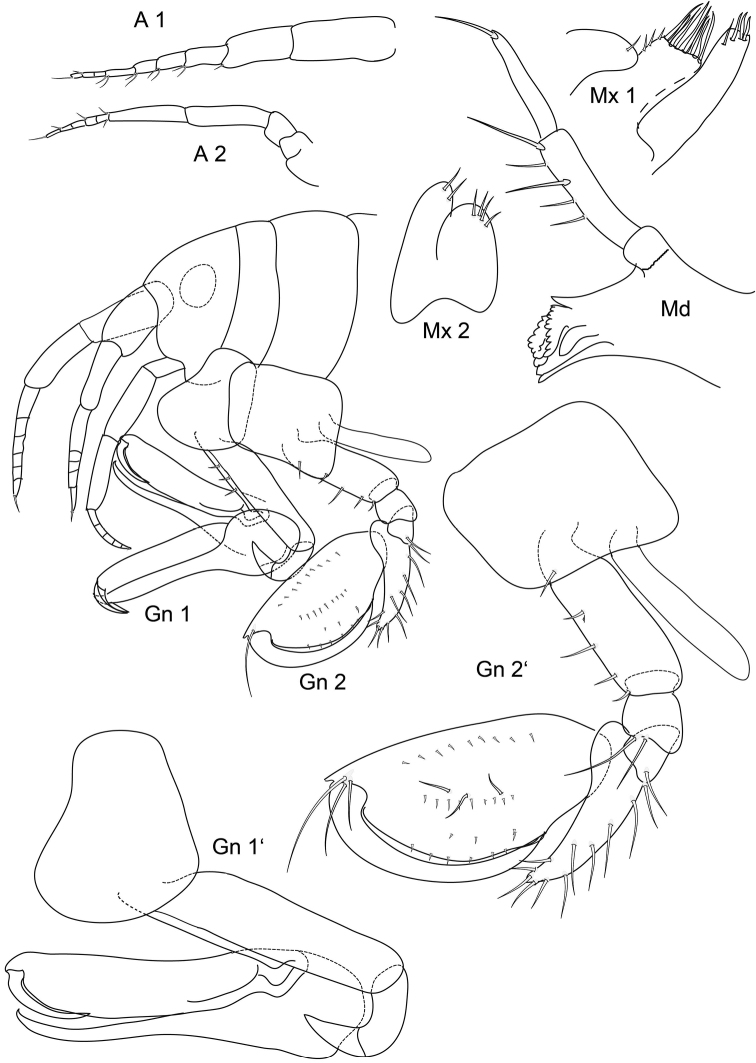
*Leucothoe vaderotti* sp. n. **A 1, A 2** antennae **Mx 1, Mx 2** maxillae **Gn 1, Gn 2** gnathopods **Gn 1', Gn 2**' gnathopods enlarged.

###### Additional material.

All in alcohol; one slide DZMB-HH52415.

DZMB–HH 52177 (ZMH K–46788): juv. 2 mm; 61°53.79'N–61°53.53'N, 010°13.77'W–010°12.65'W, Poseidon IceAGE 2, POS 456, 29.7.2013, FI Ridge, St. 878–1, 781.4–775.8 m depth, EBS Supra, 300 μm.

DZMB–HH 52021(ZMH K–46789): 1 es. 4 mm; 60°24.33'N–60°23.70'N, 006°36.91'–006°38.60'W, Poseidon IceAGE 2, POS 456, 29.7.2013, Farøer Channel South-East, St. 876–5, 554.3–674.8 m depth, shell fragments, EBS, 500 μm.

DZMB–HH 52593 (ZMHK–46790): 8 es. 3–4 mm; 61°53.79'N–61°53.53'N, 010°13.77'W–010°12.65'W, Poseidon IceAGE 2, POS 456, 29.7.2013, FI Ridge, St. 878–1, 781.4–775.8 m depth.

DZMB–HH 52617(ZMH K–46791): 1 juv. 2.5 mm; 63°42.53'N–63°42.78'N, 026°23.05'–026°22.53'W, Meteor IceAGE, ME 85–3, 9.9. 2011, South Iceland, Irminger Basin, slope, St. 1086–1, 698.1– 678.5 m depth, 730 trawling distance, EBS Supra, 300 μm.

DZMB–HH 52667 (ZMH K–46792): 1 juv. incompl., 2 mm: 61°38.50'N–61°39.24'N, 031°21.37'–031°20,95'W, Meteor IceAGE, ME 85–3, 7.9.2011, South Iceland, Irminger Basin, Deep Sea, St. 1057–1, 2504.7–2531.8 m depth, 1983 trawling distance, EBS Supra, 300 μm.

DZMB–HH 52694 (ZMH K–46793): 3 juv. 2–3 mm; 60°24.33'N–60°23.70'N, 006°36.91'–006°38.60'W, Poseidon IceAGE 2, POS 456, 29.7.2013, Farøer Channel South-East, St. 876–5, 554.3–674.8 m depth, shell fragments, EBS, 500 μm.

DZMB–HH 52415 (ZMH K–46794): 2 es. 2-3 mm; 63°42.10'N–63°42.37'N, 026°23.64'–026°23.46'W, Meteor IceAGE, ME 85–3, 9.9.2011, South Iceland, Irminger Basin, slope, St. 1082–1, 724.4–704.9 m depth, 782 trawling distance, EBS Supra, 300 μm. Slide in Faure’s medium, used for illustration.

###### Diagnosis.

Eyes oval, dark in ethanol. Mandibular palp long and narrow, art 3 more than half the length of art 2. Cx 1 inferior margin smooth, nearly as long as wide. Gn 1 carpus distal part approximately six times longer than wide, dactylus reaching approximately 1/3 of propodus length. P 3, P 4 with narrow basis, P 5 – P 7 basis oval and broadened, with regularly rounded and finely serrated hind margin. Ep 2 posterodistally with upturned tip, Ep 3 distoposterior corner with blunt, rounded angle.

###### Description.

Length 4–6 mm.

*Head.* Anterior margin rounded, anterodistal margin rectangular with rounded corner. Mid-cephalic keel with acute projection. Rostrum small.

Eyes oval. Antennae short, nearly 1/3 of body length, A 1 peduncle art 1 inferodistally with acute tooth, flagellum up to 11 arts, accessory flagellum not seen. A 2 subequal in length to A 1, peduncle art 4 > art 5, flagellum around 6 arts.

*Mouthparts.* Mandible lacking molars, palp 3-articulate, with long lateral and single distal setae. Art 3 with distal seta, incisors strongly dentate. Other mouthparts like in *L. spinicarpa*.

*Peraeon.* Cx 1– Cx 4 relative width 1: 1.2:0.8:1.

Coxa 1 smooth, length and width subequal; anterodistal margin produced, distal margin regularly rounded, facial setae absent.

Gn 1 basis not inflated, carpus distal part narrow, length to width ratio approximately 6:1; propodus straight, palm with fine short spines; dactylus curved, reaching nearly 1/3 of propodus length.

Cx 2 nearly as long as wide, subquadrangular, much wider than Cx 3, smooth; facial setae absent.

Gn 2 basis slightly inflated, on anterior margin some setae of different length; carpus reaching approx. half propodus, distally truncate, setose; propodus distally with short, sharp prolongation, palm convex, regularly rounded, with weak mediofacial setal row, with a few submarginal setae; dactylus curved, smooth, reaching more than 2/3 of propodus length.

Cx 3 length greater than its width, smooth, subrectangular with straight margins and rounded corners.

Cx 4 wider than Cx 3, posterior margin shorter than anterior one, somewhat excavate.

P 3, 4 basis narrow, approx. the width of merus; dactylus reaching or surpassing half the length of propodus.

P 5 – 7 similar, basis oval, both margins with fine serration.

*Pleon.* Ep 1 posteroventral corner rounded. Ep 2 posterodistal corner acutely produced, Ep 3 posteroventral corner bluntly rounded.

U 1 – U 3 similar, length regularly diminishing and U 2 not considerably shorter (like in *L. spinicarpa*).

**Figure 2.**
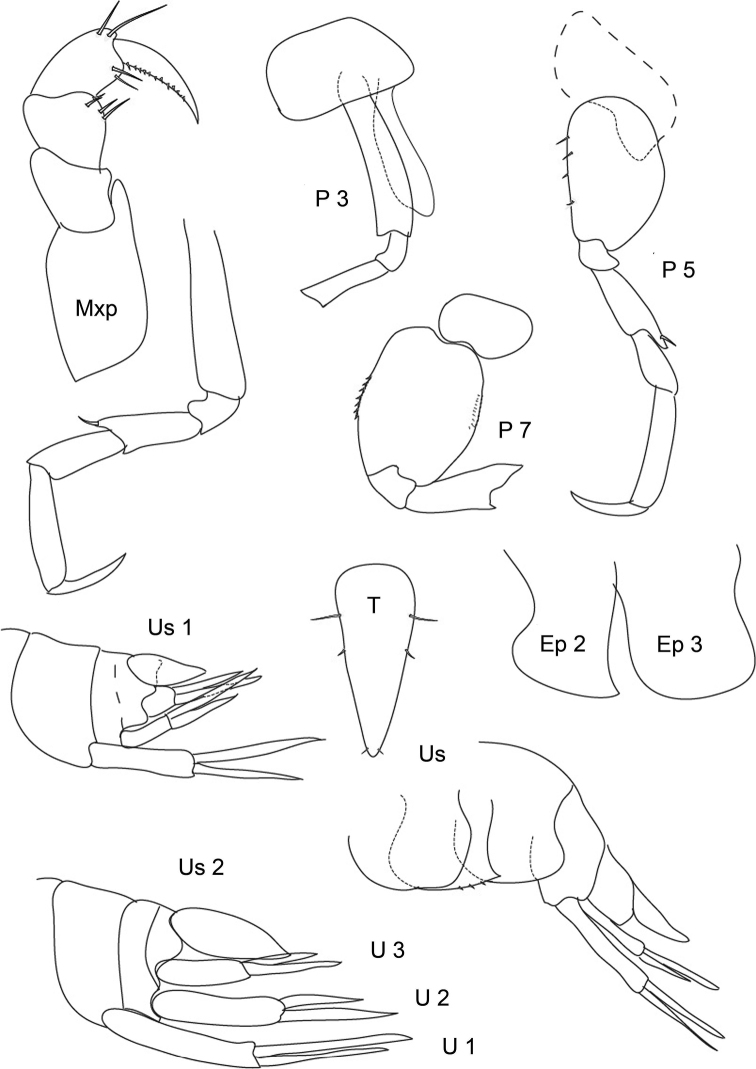
*Leucothoe vaderotti* sp. n. **Mxp** maxilliped **P 3, P 4, P 5, P 7** peraeopods **Ep 2, Ep 3** epimeral plates **Us** urosome with epimeral plates, uropods and telson **Us 1, Us 2** other urosomites **U 1, U 2, U 3** uropods **T** telson.

###### Etymology.

Wim Vader completed eight decades in February 2017. He was born Dutch but having lived for much more than half of his life in Norway, he will easily guess that I used the Norwegian word *åtti* = eighty for dedicating this Atlantic species to him. Fifty years ago we began our long-lasting and harmonious collaboration, a “golden jubilee”- thank you, Wim!

###### Geographical distribution.

South Iceland- Farøer Channel and Ridge; depth 554–2531 m.

###### Remarks.

Together with five specimens collected 1983 between Greenland and Iceland (see Krapp-Schickel and Vader 2012) the present material of the proposed new species consists of 23 animals belonging undoubtedly to the genus *Leucothoe*. All specimens are between 2 and 5 mm, rarely up to 6 mm long. There are four specimens larger than 10 mm with all characters fitting *Leucothoe spinicarpa*, sampled in similar depths as those of the animals 2–5 mm in size. The larger specimens show some differences which are not very conspicuous. It is most probable that they belong to two different species, and it seems also quite reasonable to presume that within the period of early June to end of September these 23 specimens are not all juveniles; however, no ovigerous females were found.

Differences of the small animals compared with *Leucothoe spinicarpa* are:

• Ep 3 totally blunt (see here fig. 2 and Krapp-Schickel and Vader 2012: 390 fig. 4 Ep3) vs. not rounded, but with small but clear posterodistal corner in *L. spinicarpa* (Crowe 2006: 61 fig. 1a, 63 fig. 3e; Sars 1885 pl. 101)

• U 2 in adults reaching length of U1 (see fig. 2) vs. clearly much shorter than U1 and U3 in *L. spinicarpa* (Crowe 2006 fig. 1 and fig. 4)

• Gn 1, 2 basis anterior margin with few irregular longer or shorter setae (see here fig. 1, Krapp-Schickel and Vader 2012: 389 fig. 3) vs. dense setation in *L. spinicarpa* (Crowe 2006 fig. 61 fig. 1 b–e; Sars 1895 pl. 100)

• Gn 1 dactylus less than half the length of propodus (see above fig. 1; Krapp-Schickel and Vader 2012: 389 fig. 3) vs. more than half the length of propodus in *L. spinicarpa* (Crowe 2006 fig. 61 fig 1c; Sars 1885 pl. 100)

• sizes of 23 specimens collected between 1 June–30 September are all between 2–4 mm, with only a few reaching 6 mm; vs. size range between 10–19 mm of *L. spinicarpa* (Crowe 2006, Sars 1895, Krapp-Schickel and Menioui 2005).

These differences are significant enough to distinguish these small specimens as a new species.

###### Other material examined.

Two other *Leucothoe* species were sampled, *L. spinicarpa* and *L. lilljeborgi*, the latter clearly in lower depths than the new species.

DZMB–HH 56264 (ZMH K–46795): 1 spec. 2.5 mm *Leucothoe lilljeborgi* 61°25.63'–61°25.05'N, 001°21,07'–001°21.66'E, Poseidon IceAGE 2, POS 456, 24.07.2013, Norwegian Channel, North Sea, St. 866, 169.1–168.8 m depth, EBS Epi, 500 μm.

DZMB–HH 56326 (ZMH K–46796): 2 spec. 2–2.5 mm *L. lilljeborgi* 61°25.63'–61°25.05N, 001°21.07'–001°21.66'E, Poseidon IceAGE 2, POS 456, 24.7.2013, Norwegian Channel, North Sea, St. 866, 169.1–168.8 m depth, EBS Epi, 300 μm decant.

DZMB–HH 56428 (ZMH K–46797): 1 spec. 2.5 mm *L. lilljeborgi*: 61°25.63'–61°25.05N, 001°21.07'–001°21.66'E, Poseidon IceAGE 2, POS 456, 24.7.2013, Norwegian Channel, North Sea, St. 866, 169.1–168.8 m depth, EBS Epi, 300 μm.

DZMB–HH 56500 (ZMH K–46798): 1 *L. spinicarpa* 13 mm, 2 males *L. lilljeborgi* 5.5 and 4 mm 61°59.83'–61°59.26N, 000°30.40'–000°32.32'E, Poseidon IceAGE 2, POS 456, 24.7.2013, Norwegian Channel, North Sea, St. 867, 302.5–290 m depth, EBS Epi, 500 μm.

DZMB–HH 52362 (ZMH K–46799): 1 *L. spinicarpa* 11 mm; 63°42.53'N–63°42.78'N, 026°23.05'–026°22.53'W, Meteor IceAGE, ME 85-3, 9.9.2011, South Iceland, Irminger Basin, slope, St. 1086-1, 698,1–678.5 m depth, 730 trawling distance, EBS Supra, 300 μm.

DZMB–HH 52627 (ZMH K–46800): 1 *L. spinicarpa* 8 mm 62°33.10'N–62°33.22'N, 020°23.71'–020°22,87'W, Meteor IceAGE, ME 85-3, 2.9.2011, South Iceland, Iceland Basin, slope, St. 1010–1, 1384.8–1389 m depth, 1183 trawling distance, EBS Supra, 300 μm.

DZMB–HH 32864 (ZMH K–46801): 1 juv. *Leucothoe* sp. imperf. 2 mm, together with *Leucothoe spinicarpa* 12 mm; 61°53.79'N–61°53.53'N, 010°13.77'W–010°12.65'W, Poseidon IceAGE 2, POS 456, 29.7.2013, FI Ridge, St. 878-1, 781.4–775.8 m depth, EBS Supra, 300 μm.

## Discussion

After Krapp-Schickel and Vader (2012) many *Leucothoe* species are known from the Atlantic Ocean, but mainly from warmer regions. Not much is known about their biology, but it is known that they prefer to live near, in, or with other species such as sponges, and this may also be the reason that they are often well hidden and thus overlooked when generalised ship sampling occurs.

**Table:** 

Locality	Species	Author
West Africa	*Leucothoe minima*	Schellenberg, 1925
	*L. occidentalis*	Reid, 1951
	*L. brunonis*	Krapp-Schickel & Menioui, 2005
	*L. campi*	Mateus & Mateus, 1986
	*L. spinulosa*	Chevreux, 1920
South Africa	*L. miersi*	Stebbing, 1888
	*L. ctenocheir*	K.H. Barnard, 1925
	*L. dolichoceras*	K.H. Barnard, 1916
Caribbean, Gulf of Mexico	*L. wuriti*	Thomas & Klebba, 2007
	*L. flammosa*	Thomas & Klebba, 2007
	*L. hortapugai*	Winfried et al. 2009
	*L. barana*	Thomas & Klebba, 2007
	*L. ortizi*	Winfried et al. 2009
	*L. hendrickxi*	Winfried et al. 2009
	*L. saron*	Thomas & Klebba, 2007
	*L. ubouhu*	Thomas & Klebba, 2006
	*L. garigunae*	Thomas & Klebba, 2007
Brazil	*L. cheiriserra*	Serejo, 1998
	*L. lihue*	Barnard, 1970
	*L. basilobata*	Serejo, 1998
	*L. urospinosa*	Serejo, 1998
	*L. leptosa*	Serejo, 1998
Biscaya and Azores	*L. cathalaa*	Frutos & Sorbe, 2012
	*L. rostrata*	Chevreux, 1908
Barbados, Mid Atlantic ridge	*L. ayrtonia*	Bellan-Santini, 1997
	*L. atosi*	Bellan-Santini, 1997
Great Britain, Scotland, northern Atlantic	*L. incisa*	Robertson, 1892
	*L. procera*	Bate, 1857
	*L. lilljeborgi*	Boeck, 1861
	*L. richiardii*	Lessona, 1865
	*L. spinicarpa*	Abildgaard, 1789
	*L. articulosa*	(Montagu, 1804)
	*L. uschakovi*	Gurjanova, 1951

## Supplementary Material

XML Treatment for Leucothoe vaderotti
